# Retrospective evaluation of ocular injuries in fractures of the zygomaticoorbital complex in a level I trauma center: is primary specialized ophthalmologic examination always necessary?

**DOI:** 10.1007/s10792-025-03566-7

**Published:** 2025-05-17

**Authors:** Vanessa Kramer, Efstathios Vounotrypidis, Christian Steinkohl, Christel Weiß, Alexander Schramm, Frank Wilde, Marcel Ebeling, Andreas Sakkas

**Affiliations:** 1 Department of Oral and Maxillofacial Surgery, University Hospital Ulm, Ulm, Germany; 2 Department of Oral and Plastic Maxillofacial Surgery, Military Hospital Ulm, Ulm, Germany; 3 Department of Ophthalmology, University Hospital Ulm, Ulm, Germany; 4 Department of Ophthalmology, Military Hospital Ulm, Ulm, Germany; 5 Medical Statistics and Biomathematics, University Medical Centre Mannheim, Heidelberg University, Mannheim, Germany

**Keywords:** Zygomaticoorbital fracture, Orbital fracture, Ocular injury, Ophthalmologic evaluation

## Abstract

**Purpose:**

The necessity of a specialized ophthalmological assessment following fractures of the zygomaticoorbital (ZMO) complex in an emergency setting is still debated in resource-limited health systems. The primary aim of this study was to determine the incidence and types of ocular and periocular injuries (OPIs) associated with different fracture patterns of the ZMO complex. The secondary aim was to investigate the association between patient- and trauma-specific variables with the different types of OPI and identify high-risk patients for severe OPI.

**Methods:**

A retrospective cohort study was conducted on patients with ZMO complex fractures over a seven-year period. All patients underwent a specialized ophthalmic assessment in the ophthalmology clinic within 24 h of initial admission. Visual acuity, extraocular eye movements, and pupillary reaction were examined to determine the type of OPI that occurred. Demographic and medical history data, clinical and radiological findings and specific OPIs were recorded. Demographics, fracture patterns and ophthalmological findings were presented using descriptive statistical analysis. A multivariable analysis was performed to identify associations between predictor factors (etiology of injury, fracture pattern, pre-traumatic ophthalmological conditions, antithrombotic therapy) and OPI severity.

**Results:**

489 patients with a mean age of 50.88 years and a total of 540 examined eyes met the inclusion criteria. Ground-level fall was the most common etiology of injury. Periocular hematomas (28.99%), subconjunctival hemorrhage (18.65%) and periorbital swelling (15.13%) were the most common ophthalmologic findings. Eye motility disorders (p = 0.0003) and diplopia (p = 0.0019) were significantly more common in isolated orbital wall fractures than in other midface fracture patterns. Chemosis was significantly more common in fractures of the zygomaticoorbital complex (p = 0.0199), while lid tears (p = 0.0470) and open globe injuries (p = 0.0002) were more common in Le Fort fractures. Optic disc hemorrhage occurred significantly more frequently in patients under single antithrombotic therapy (p = 0.0171). Blow from blunt objects and Le Fort fractures were associated with higher rates of severe OPI, while pre-traumatic ophthalmologic conditions and antithrombotic therapy were not.

**Conclusions:**

Within the limitations of the study, patients who experienced blows from blunt objects and those with Le Fort fractures are at a higher risk for severe OPIs. Early specialized ophthalmological consultation is recommended for patients with zygomatic fractures and orbital involvement, especially for those with visual alterations such as motility disorders and diplopia as well as those taking antithrombotic medication.

## Introduction

Trauma to the zygomaticoorbital (ZMO) complex accounts for approximately 27% of all facial fractures making it a significant concern in cranio-maxillofacial surgery [1–3]. Fractures involving the orbital wall are often linked to ocular and periocular injuries (OPI), which occur in up to 94% of cases [4–9]. Scruggs et al. observed an increase in ocular trauma cases over a five-year period, influenced by different populations [10]. Steinberg et al. reported that most eye-related hospital admissions are due to trauma, with 90% of them being preventable [11].

The severity of OPI depends on various factors such as the mechanism of trauma, fracture pattern, location and complexity [8]. The occurrence of severe OPI requiring urgent intervention ranges from 2.7 to 13.7%, considered relatively low [11, 12]. Different authors have varying criteria for defining severity, with some focusing on permanent sequelae and others on the need for urgent ophthalmic surgery [9]. The studies of Rossin et al. and Richani et al. have identified several risk factors for severe injury such as diplopia, fracture location, pain with eye movements, blurry vision, and motility restriction, however, they differ regarding which factors are considered the most prognosticative [13, 14]. Thus, there is no standardized classification system for assessing the severity of OPI.

Prompt identification and proper management of visual and functional deficits can help preserve visual function and prevent long-term complications [1–4, 7, 9]. The decision to seek specialized ophthalmological assessment is an essential dilemma in emergency departments. As health resources are limited and consultation is not always possible, especially in hospitals without a 24-h ophthalmology specialist, decision-making remains subject to the physician’s individual judgment. Ross et al. and Mellema et al. suggested in their studies that routine cases of orbital fractures in the absence of suspicion of serious ocular injury and visually asymptomatic patients may be less likely to have pathologies requiring emergent evaluation by an ophthalmologist [12, 15]. Other authors consider the ophthalmological status essential for any periorbital trauma and suggest an emergent ophthalmologic consultation regardless of the clinical signs and the indication for fracture repair, since ophthalmologists can detect minor ocular injuries that might otherwise go unnoticed [3, 7]. The timing of the ophthalmological assessment is also debatable; some surgeons recommend fracture treatment based on ophthalmic symptoms at admission, while others prefer to wait for an ophthalmological reassessment after regression of soft tissue swelling and re-evaluate the need for surgical intervention regardless of fracture dislocation grade [16, 17].

At many institutions, including ours, multiple specialists including oral and maxillofacial surgeons, otolaryngologists, ophthalmologists and general practitioners collaborate to optimize outcomes for patients with fractures of the ZMO complex and associated OPI. The time until the ophthalmological consultation is multifactorial and depends on the referring physician, the time of day, and the contacted ophthalmologist. However, reflexive, emergent ophthalmology consultation for every fracture could place a significant burden on the healthcare system, resulting in many normal exams, especially considering the variable incidence of OPI. A more practical approach would be to implement triage systems for OPI, managed by the maxillofacial team or emergency physicians. Previous research has attempted to develop simple scoring systems or identify predictors that can be promptly applied at the bedside by non-ophthalmologists, facilitating effective triage and management [5, 6]. However, these systems or predictors have never been validated by prospective multicenter studies and thus remain an ideal but elusive tool for most practitioners managing patients with ZMO trauma [9]. There is a lack of knowledge regarding the necessity for an urgent ophthalmological consultation for every ZMO trauma case, independent of the fracture pattern and indication for primary fracture management.

The primary aim of this study was to determine the incidence and type of OPI associated with different fracture patterns of the ZMO complex. The secondary aim was to investigate the association between patient- and trauma-specific variables with the different types of OPI and identify high-risk patients for severe OPI. We also aimed to determine whether an ophthalmological consultation is always required in an emergency setting, and if so, in which cases. This knowledge could be crucial in health centers without in-house ophthalmology specialists and thus guide prompt identification and adequate management decisions.

## Methods

### Study design and ethics

To address the research objectives, the authors designed and implemented a retrospective cohort study. Medical records of patients with various fracture patterns of the ZMO complex who were primarily treated in our clinic of Oral and Plastic Maxillofacial Surgery between January 2016 and March 2023 were reviewed. Records were retrieved from the hospital electronic database. Ethical approval for this study was obtained from the ethics committee of the Chamber of Physicians in Baden-Württemberg, University of Ulm, Germany (approval number: 102/23, date of approval: 05.04.2023). The study was designed according to the recommendations of the Strengthening the Reporting of Observational Studies in Epidemiology (STROBE) guideline and was performed in accordance with the Declaration of Helsinki 1964 and its later amendments (World Medical Association, Declaration of Helsinki) [18].

### Participants

Patients who met the following inclusion criteria were enrolled: [1] patients of any age with any etiology of injury, [2] patients who underwent a head CT scan after blunt trauma of any etiology, [3] radiologically diagnosed zygomatic fractures without orbital floor involvement, [4] radiologically diagnosed zygomatic fractures with orbital floor involvement, [5] radiologically diagnosed single-wall or multiple-wall orbital fractures (orbital floor, medial wall, lateral wall, orbital rim), [6] Le Fort fractures (II, III), and [7] primary clinical and ophthalmological assessment by both maxillofacial surgeons and ophthalmologists within 24 h after initial admission.

The exclusion criteria were: [1] midface fractures not consistent with those mentioned above, [2] patients with previous monocular or non-stereoscopic vision, [3] cases without consultation by ophthalmology specialists, [4] cases with delayed untreated fractures, [5] cases in which the primary ophthalmological evaluation did not take place in our institution, and [6] incomplete medical records.

### Patient screening

Codes from the ICD-10-GM diagnostic manual were utilized to identify patients presenting with the aforementioned fracture patterns. The patient case number was then used to search for the electronic medical records in the digital patient information system (Nexus®, Nexus AG, Donaueschingen, Germany). The clinical and radiological findings from the Clinic of Oral and Plastic Maxillofacial Surgery and clinic of Ophthalmology, as well as the in-hospital admission and/or discharge documents were used for data collection.

### Protocol in the emergency department

After an initial evaluation at the emergency department, a maxillofacial surgeon conducted the primary clinical and ophthalmological examination. This examination included taking a medical history, inspecting the midface, lower face and peri-orbital region, conducting a gross inspection of the eye globe, and assessing the size and reactivity of the pupils. Additionally, assessments were made for gross visual acuity, extraocular eye movements (EOMs), diplopia, enophthalmos/exophthalmos and neuro-sensory disturbances of the infraorbital nerve using a two-point discrimination test. Following the initial examination, a definitive diagnosis was made by performing 3D imaging using a CT scan. The radiological data used for this diagnosis were obtained from existing radiology reports written by board-certified radiologists.

### Specialized ophthalmological evaluation

Afterward, all patients were referred for specialized consultation with ophthalmological colleagues within 24 h of admission. This consultation could involve either an ophthalmology resident and an attending ophthalmologist, or just an attending alone.

The ophthalmological examination for diagnosing ocular injuries during regular working hours included a visual acuity test, intraocular pressure (IOP) measurement, eyelid, conjunctival, corneal, and pupil examination, slit lamp microscopy of the anterior segment of the eye, and fundus examination in miosis. Additional examinations included tonometry, extraocular muscles, and visual field testing. Outside regular working hours, the examination included a visual acuity test, IOP measurement, eyelid, conjunctival, corneal and pupil examination and slit lamp microscopy of the anterior segment. A dilated fundus examination was not included in the specialized ophthalmological assessment unless there was high suspicion of intraocular involvement to avoid damage to the iris sphincter. This examination was performed 7–10 days post-trauma. In cases of posterior segment pathology related to the trauma, a fundus examination in mydriasis was carried out directly. Further examinations such as ultrasound, macular OCT, and optic disc OCT, were performed as necessary based on clinical findings.

For patients with polytrauma and accompanying injuries in other parts of the body who were admitted intubated and ventilated to our interdisciplinary emergency department, the ophthalmological examination focused on ruling out an open globe injury and retrobulbar hematoma. Visual acuity testing and eye muscle motility testing were not conducted.

### Data collection.

All patients were anonymized before data analysis. The data were collected from patients’ hospital charts and included demographics, etiology of injury, previous ophthalmologic pathologies, antithrombotic medication, CT findings (including fracture characteristics such as pattern, location, and number of orbital walls involved), and ophthalmologic findings at the initial examination.

### Study variables

The predictor variables included:

1. Etiology of injury.
2. Fracture patterns of the ZMO complex:
  (A) Zygomatic fracture without orbital floor involvement.
  (B) Zygomatic fracture with orbital floor involvement.
  (C) Isolated single-wall or multiple-wall orbital fractures (orbital floor, medial wall, lateral wall, orbital rim).
  (D) Le Fort fractures (II, III).
3. Pre-traumatic ophthalmologic conditions:
  (A) Glaucoma (grade not considered).
  (B) Cataract (grade not considered).
  (C) Previous eye surgery.
4. Antithrombotic therapy:
  (A) Single medication:
    - Single antiplatelet agent users (acetylsalicylic acid, clopidogrel, prasugrel, and ticagrelor).
    - Direct oral anticoagulant (DOAC) users (apixaban, rivaroxaban, edoxaban and dabigatran).
    - Vitamin K antagonist (VKA) users (phenprocoumon).
  (B) Double medication (dual antiplatelet therapy or a combination of an antiplatelet agent and an anticoagulant from any other group).

The primary outcome was OPI, categorized as follows: [1] severe if immediate surgical treatment was necessary, and [2] non-severe if it was not. Immediate intervention was considered as a procedure performed without delay or within six hours, with the main objective of preventing severe visual impairment or blindness.

### Statistical analysis

Data was centralized in electronic format using Microsoft Excel software and analyzed descriptively. Statistical analysis was performed using SAS®, Release 9.4 software (SAS Institute Inc., Cary NC, USA). Descriptive statistics were used to describe baseline patient characteristics. All categorical variables were expressed as absolute values (n) and relative incidences (%). For metric variables, the standard deviation was calculated. The incidence of different fracture patterns and OPIs were determined. The Kolmogorov–Smirnov test revealed an abnormal distribution of the data. Associations between categorical variables were described by cross-tabulations, and a multivariable analysis with chi-square tests was used to investigate a potential association between the predictor variables and OPIs. Fisher’s exact test with Monte-Carlo-simulation was used to compare smaller subgroups. A multimodal bivariate analysis was conducted to find an association between the predictor variables and severity of OPI. The significance level was set at two-sided p ≤ 0.05.

## Results

### Demographic distribution

A total of 489 patients with 540 eyes met the inclusion criteria, with a mean age of 50.88 ± 22.03 years (range: 11–94 years) at the time of injury and a male predominance (n = 335/489; 68.51%; male:female ratio = 2.17:1). The most affected age group was between 21 and 30 years (n = 105; 21.47%). When considering age distribution by gender, the highest frequency for male patients was in the second decade of life (n = 91; 18.61%), while the most common age for injury among female patients was between 71 and 80 years (n = 34; 6.95%).

### Etiology of injury

Out of the total number of evaluated eyes (n = 540), ground-level falls were the most common etiology of injury (n = 171, 31.67%), followed by violence (n = 104; 19.26%) and bicycle accidents (n = 102; 18.89%). Road traffic accidents were less common, accounting for 5.37% (n = 29) of the cases.

### Fracture patterns

**Fig. 1:**
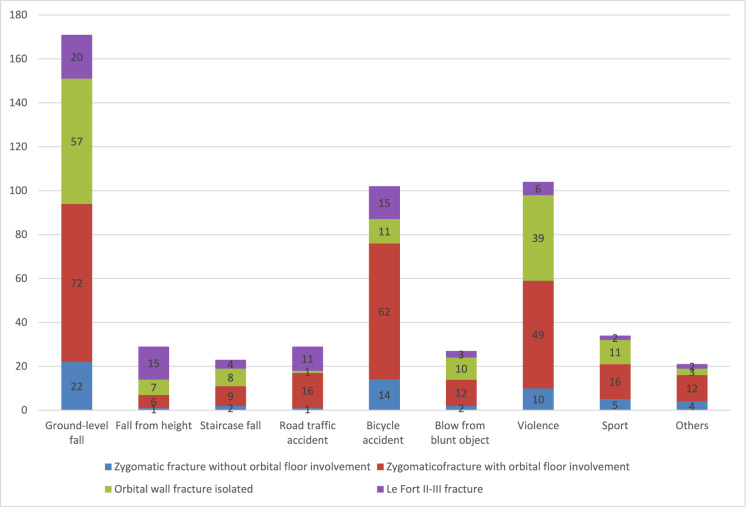
Distribution of the various fracture patterns in relation to the etiology of injury (n = 540 trauma-related eyes examined)

During the study period, a total of 438 (89.57%) unilateral and 51 (10.43%) bilateral fractures were recorded. The most common fractures were of the ZMO complex (n = 254; 47.04%), followed by isolated orbital wall fractures (n = 147; 27.22%), Le Fort fractures (n = 78; 14.44%), and zygomatic fractures without orbital involvement (n = 61; 11.30%). The distribution of the various fracture patterns in relation to the etiology of injury is presented in Fig. 1. Among the orbital wall fractures, single orbital wall fractures were most common (n = 131; 89.11%), with fewer patients having two (n = 13; 8.84%) or three (n = 3; 2.04%) walls fractured. Of the single orbital wall fractures, orbital floor fractures were the most prevalent (n = 126; 85.71%), followed by medial (n = 3; 2.04%), lateral (n = 1; 0.68%) and roof (n = 1; 0.68%) fractures.

### Ophthalmologic examination

A total of 1421 ophthalmologic diagnoses were recorded (Table 1).

**Table 1:** Distribution of ophthalmologic examination findings in relation to various fractures patterns (n = 1421)

Ophthalmologic examination findings	Zygomatic fracture without orbital floor involvement	Zygomatic fracture with orbital floor involvement	Orbital wall fracture isolated	Le Fort II-III fracture	n (%)
Non-severe					
Periocular hematoma	46 (3.24%)	200 (14.07%)	116 (8.16%)	50 (3.52%)	412 (28.99%)
Hyposphagma	28 (1.97%)	135 (9.50%)	73 (5.14%)	29 (2.04%)	265 (18.65%)
Periorbital swelling	29 (2.04%)	101 (7.11%)	65 (4.57%)	20 (1.41%)	215 (15.13%)
Eye motility disorder*	12 (0.84%)	63 (4.43%)	75 (5.28%)	9 (0.63%)	159 (11.19%)
Diplopia**	10 (0.70%)	58 (4.08%)	64 (4.50%)	7 (0.49%)	139 (9.78%)
Chemosis***	5 (0.35%)	28 (1.97%)	20 (1.41%)	15 (1.06%)	68 (4.79%)
Pupil difference	0	11 (0.77%)	8 (0.56%)	2 (0.14%)	21 (1.48%)
Subjective eye pain	3 (0.21%)	9 (0.63%)	5 (0.35%)	2 (0.14%)	19 (1.34%)
Ptosis	4 (0.28%)	5 (0.35%)	3 (0.21%)	2 (0.14%)	14 (0.99%)
Emphysema	4 (0.28%)	3 (0.21%)	5 (0.35%)	2 (0.14%)	14 (0.99%)
Increased intraocular pressure	0	5 (0.35%)	6 (0.42%)	2 (0.14%)	13 (0.91%)
Corneal abrasion	1 (0.07%)	6 (0.42%)	5 (0.35%)	0	12 (0.84%)
Enophthalmos	0	4 (0.28%)	5 (0.35%)	0	9 (0.63%)
Visus acuity change	1 (0.07%)	4 (0.28%)	4 (0.28%)	0	9 (0.63%)
Macular edema	1 (0.07%)	3 (0.21%)	4 (0.28%)	0	8 (0.56%)
Exophthalmos	0	5 (0.35%)	1 (0.07%)	0	6 (0.42%)
Ectropion	0	2 (0.14%)	1 (0.07%)	2 (0.14%)	5 (0.35%)
Optic disc hemorrhage	0	2 (0.14%)	1 (0.07%)	1 (0.07%)	4 (0.28%)
Anterior uveitis	0	1 (0,07%)	1 (0.07%)	0	2 (0.14%)
Nystagmus	0	1 (0.07%)	0	0	1 (0.07%)
Visual field restriction	0	1 (0.07%)	0	0	1 (0.07%)
Entropiom	0	1 (0.07%)	0	0	1 (0.07%)
Lagophthalmos	0	1 (0.07%)	0	0	1 (0.07%)
Iris swelling	0	1 (0.07%)	0	0	1 (0.07%)
Optic nerve congestion	0	0	0	1 (0.07%)	1 (0.07%)
Hyphema	0	0	0	1 (0.07%)	1 (0.07%)
Severe					
Lid tear****	0	1 (0.07%)	2 (0.14%)	3 (0.21%)	6 (0.42%)
Open globe injury*****	0	0 (0.00%)	0	4 (0.28%)	4 (0.28%)
Retrobulbar hematoma	0	2 (0.14%)	1 (0.07%)	0	3 (0.21%)
Macular hemorrhage	1 (0.07%)	2 (0.14%)	0	0	3 (0.21%)
Foreign body	0	2 (0.14%)	1 (0.07%)	0	3 (0.21%)
Lens subluxation	0	1 (0.07%)	0	0	1 (0.07%)
n (%)	145 (10.20%)	658 (46.31%)	466 (32.79%)	152 (10.70%)	1421 (100%)

*n* = number; *%* = percentage

*Chi2-test: **p = 0.0003**: Eye motility disorders occurred significantly more frequently in isolated orbital wall fractures than in other fracture patterns

**Chi2-test: **p = 0.0019**: Diplopia occurred significantly more frequently in isolated orbital wall fractures than in other fracture patterns

***Chi2-test: **p = 0.0199**: Chemosis occurred significantly more frequently in zygomatic fractures with orbital involvement than in other fracture patterns

****Fisher’s Exact Test: **p = 0.0470**: Eye lid tears occurred significantly more frequently in Le Fort fractures than in other fracture patterns

*****Fisher’s Exact Test: **p = 0.0002**: Ruptured globe occurred significantly more frequently in Le Fort fractures than in other fracture patterns

The most common diagnosed finding was periocular hematoma (n = 412; 28.99%), followed by hyposphagma (n = 265; 18.65%) and periorbital swelling (n = 215; 15.13%). Motility disorders accounted for 11.19% (n = 159) and diplopia for 9.78% (n = 139) of all ophthalmologic findings. Nystagmus, visual field restriction, entropion, lens subluxation, lagophthalmos, papillary swelling, optic nerve congestion and hyphema were each recorded in one patient case (0.07%). Three cases of retrobulbar hematomas (0.21%) were documented.

Motility disorders (n = 75; p = 0.0003) and diplopia (n = 64; p = 0.0019) were significantly more common in orbital wall fractures compared to other fracture patterns (Table 1). Patients with isolated orbital floor fractures were particularly affected by these two ophthalmological findings (motility disorders n = 66; diplopia n = 55). For zygomatic fractures with orbital involvement, a significant association was only found for post-traumatic chemosis (n = 28; p = 0.0199). Eyelid tears (n = 3; p = 0.0470) and open-globe injuries (n = 4; p = 0.0002) were recorded more frequently in Le Fort fractures. When classifying OPI into open-globe and closed-globe injuries, only four open globe injuries (0.28%) occurred. No statistical correlations were found for zygomatic fractures without orbital floor involvement and other ophthalmological findings (Table 1). No association between ophthalmological findings and orbital wall fractures was found (p = 0.8276) (Table 2).

**Table 2:** Distribution of ophthalmologic examination findings related to the orbital wall(s) fractures

Ophthalmologic examination findings	Orbital wall(s) fracture(s)											p value
	a	b	c	d	a + b	a + c	a + d	a + b + c	a + b + c	c + d		
	n (%)	n (%)	n (%)	n (%)	n (%)	n (%)	n (%)	n (%)	n (%)	n (%)	n (%)	
Severe												0.8276*
Periocular hematoma	98 (21.03%)	1 (0.21%)	1 (0.21%)	1 (0.21%)	6 (1.29%)	2 (0.43%)	3 (0.64%)	1 (0.21%)	2 (0.43%)	1 (0.21%)	116 (24.89%)	
Hyposphagma	62 (13.30%)	2 (0.43%)	1 (0.21%)	1 (0.21%)	3 (0..64%)	0	2 (0.43%)	1 (0.21%)	1 (0.21%)	0	73 (15.67%)	
Periorbital swelling	58 (12.45%)	1 (0.21%)	1 (0.21%)	0	1 (0.21%)	2 (0.43%)	1 (0.21%)	0	1 (0.21%)	0	65 (13.95%)	
Eye motility disorder	66 (14.16%)	1 (0.21%)	0	0	4 (0.86%)	2 (0.43%)	1 (0.21%)	0	1 (0.21%)	0	75 (16.09%)	
Diplopia	55 (11.80%)	1 (0.21%)	0	0	3 (0.64%)	1 (0.21%)	0	1 (0.21%)	2 (0.43%)	1 (0.21%)	64 (13.73%)	
Chemosis	17 (3.65%)	0	0	0	2 (0.43%)	1 (0.21%)	0	0	0	0	20 (4.29%)	
Pupil difference	7 (1.50%)	0	0	0	1 (0.21%)	0	0	0	0	0	8 (1.72%)	
Subjective eye pain	5 (1.07%)	0	0	0	0	0	0	0	0	0	5 (1.07%)	
Ptosis	3 (0.64%)	0	0	0	0	0	0	0	0	0	3 (0.64%)	
Emphysema	3 (0.64%)	0	0	1 (0.21%)	0	0	1 (0.21%)	0	0	0	5 (1.07%)	
Increased intraocular pressure	6 (1.29%)	0	0	0	0	0	0	0	0	0	6 (1.29%)	
Corneal abrasion	4 (0.86%)	0	0	0	1 (0.21%)	0	0	0	0	0	5 (1.07%)	
Enophthalmos	3 (0.64%)	1 (0.21%)	0	0	0	1 (0.21%)	0	0	0	0	5 (1.07%)	
Visus reduction	2 (0.43%)	1 (0.21%)	0	0	1 (0.21%)	0	0	0	0	0	4 (0.86%)	
Macular edema	3 (0.64%)	0	0	0	0	0	0	1 (0.21%)	0	0	4 (0.86%)	
Exophthalmos	1 (0.21%)	0	0	0	0	0	0	0	0	0	1 (0.21%)	
Ectropion	1 (0.21%)	0	0	0	0	0	0	0	0	0	1 (0.21%)	
Optic disc hemorrhage	1 (0.21%)	0	0	0	0	0	0	0	0	0	1 (0.21%)	
Anterior uveitis	1 (0.21%)	0	0	0	0	0	0	0	0	0	1 (0.21%)	
Non-severe												
Lid tear	2 (0.43%)	0	0	0	0	0	0	0	0	0	2 (0.43%)	
Retrobulbar hematoma	1 (0.21%)	0	0	0	0	0	0	0	0	0	1 (0.21%)	
Foreign body	1 (0.21%)	0	0	0	0	0	0	0	0	0	1 (0.21%)	
n (%)	400 (85.84%)	8 (1.72%)	3 (0.64%)	3 (0.64%)	22 (4.72%)	9 (1.93%)	8 (1.72%)	4 (0.86%)	7 (1.50%)	2 (0.43%)	466 (100%)	

*a* Orbital floor, *b* medial orbital wall, *c* lateral orbital wall, *d* orbital roof

*Fisher’s exact test with Monte-Carlo-simulation

When classifying OPIs into non-severe and severe, 20 (1.4%) severe injuries occurred, which were immediately addressed surgically (Table 1).

Adnexal injuries were classified as eyelid injuries and conjunctiva injuries, while orbital injuries were described in the fracture patterns section separately. Among eyelid injuries, periocular hematoma was the most common (n = 412; 28.99%), followed by ptosis (n = 14; 0.99%). Conjunctival injuries were represented by hyposphagma (n = 165; 18.65%) and chemosis (n = 68; 4.79%).

### Pre-traumatic ophthalmological conditions

In the medical history of the 112 patients surveyed, 22.90% reported previous ophthalmological diseases and/or operations. The most common condition reported was cataract with 61.61% (n = 69) of patients indicating this, followed by previous eye surgery (n = 25; 22.32%) and glaucoma (n = 13; 11.61%). Combinations of these conditions were rare. The statistical analysis did not reveal any significant associations between previous ophthalmological conditions and the findings of the ophthalmological examination (Table 3).

**Table 3:** Distribution of ophthalmologic examination findings in patients with pre-existing ophthalmic conditions before trauma (n = 126)

Ophthalmologic examination findings	Glaucoma	Cataract	Previous eye surgery	Glaucoma + Cataract	Glaucoma + Previous eye surgery	Cataract + Previous eye surgery	n (%)
Non-severe							
Hyposphagma*	5 (3.97%)	32 (25.40%)	10 (7.94%)	0	2 (1.59%)	1 (0.79%)	50 (39.68%)
Eye motility disorder*	1 (0.79%)	22 (17.46%)	8 (6.35%)	1 (0.79%)	0	1 (0.79%)	33 (26.19%)
Diplopia*	1 (0.79%)	18 (14.29%)	10 (7.94%)	0	0	1 (0.79%)	30 (23.81%)
Pupil difference*	0	3 (2.38%)	3 (2.38%)	0	0	0	6 (4.76%)
Macular edema	1 (0.79%)	1 (0.79%)	0	0	0	0	2 (1.59%)
Corneal abrasion	0	0	1 (0.79%)	0	0	0	1 (0.79%)
Visual field restriction	0	0	1 (0.79%)	0	0	0	1 (0.79%)
Optic disc hemorrhage	1 (0.79%)	0	0	0	0	0	1 (0.79%)
Severe							
Macular hemorrhage	1 (0.79%)	1 (0.79%)	0	0	0	0	2 (1.59%)
n (%)	10 (7.94%)	77 (61.11%)	33 (26.19%)	1 (0.79%)	2 (1.59%)	3 (2.38%)	126 (100%)

*n* = number; % = percentage

*Fisher’s exact test: **p > 0.05**

### Antithrombotic therapy

Periocular hematomas were most frequently recorded in patients with single antithrombotic therapy (n = 67; 27.69%). Among them, the most common were in patients treated with antiplatelet agents (n = 45; 18.60%). Hyposphagma occurred in 40 cases (16.53%) and periorbital swelling in 38 cases (15.70%) respectively, with the majority receiving single therapy with antiplatelet agents. No significant association was found between ophthalmologic findings and antithrombotic medications (Table 4).

**Table 4:** Distribution of ophthalmologic examination findings in patients undergoing therapy with various antithrombotic medications (n = 242)

Ophthalmologic examination findings	Antiplatelet agents	Direct oral anticoagulants	Vitamin K antagonists	Dual antiplatelet agents	Antiplatelet agents + DOACs	
Non-severe						
Periocular hematoma*	45 (18.60%)	16 (6.61%)	6 (2.48%)	1 (0.41%)	1 (0.41%)	69 (28.51%)
Hyposphagma*	24 (9.92%)	12 (4.96%)	4 (1.65%)	2 (0.83%)	1 (0.41%)	43 (17.77%)
Periorbital swelling*	25 (10.33%)	10 (4.13%)	3 (1.24%)	2 (0.83%)	1 (0.41%)	41 (16.94%)
Eye motility disorder*	14 (5.79%)	3 (1.24%)	5 (2.07%)	1 (0.41%)	0	23 (9.50%)
Diplopia*	13 (5.37%)	6 (2,48%)	4 (1.65%)	1 (0.41%)	0	24 (9.92%)
Chemosis*	12 (4.96%)	4 (1.65%)	1 (0.41%)	0	0	17 (7.02%)
Pupil difference	0	0	1 (0.41%)	0	0	1 (0.41%)
Subjective eye pain	2 (0.83%)	0	0	0	0	2 (0.83%)
Ptosis*	4 (1.65%)	0	0	0	0	4 (1.65%)
Emphysema*	1 (0.41%)	1 (0.41%)	1 (0.41%)	0	0	3 (1.24%)
Increased intraocular pressure	1 (0.41%)	1 (0.41%)	0	0	0	2 (0.83%)
Corneal abrasion	0	0	0	0	0	0 (0.00%)
Enophthalmos	1 (0,41%)	0	0	0	0	1 (0.41%)
Visus acuity change	1 (0.41%)	0	0	0	0	1 (0.41%)
Macular edema	0	1 (0.41%)	0	0	0	1 (0.41%)
Exophthalmos	1 (0.41%)	1 (0.41%)	0	0	0	2 (0.83%)
Ectropion	0	1 (0.41%)	0	0	0	1 (0.41%)
Optic disc hemorrhage*	2 (0.83%)	1 (0.41%)	0	0	0	3 (1.24%)
Pupillary swelling	1 (0.41%)	0	0	0	0	1
Severe						
Retrobulbar hematoma	0	1 (0.41%)	0	0	0	1 (0.41%)
Macular hemorrhage	2 (0.83%)	0	0	0	0	2 (0.83%)
n (%)	149 (61.57%)	58 (23.97%)	25 (10.33%)	7 (2.89%)	3 (1.24%)	242 (100%)

*n* = number; % = percentage

*Fisher’s exact test: **p > 0.05**

Hyposphagma and periorbital swelling occurred most frequently in patients receiving dual antithrombotic therapy (n = 3; 1.24%, respectively). Hyposphagma was recorded in two (0.83%) patients with dual antiplatelet therapy and in one (0.41%) with a combination of an antiplatelet agent and DOAC. Peribulbar hematoma was diagnosed in one patient (0.41%) under dual antiplatelet therapy and one patient (0.41%) with a combination of an antiplatelet agent and DOAC. Comparing ophthalmologic findings in patients on single or double antithrombotic therapy, optic disc hemorrhage occurred significantly more frequently in patients with single antithrombotic therapy (p = 0.0171) (Table 5).

**Table 5:** Distribution of ophthalmologic examination findings in patients under single and double antithrombotic therapy compared to patients without antithrombotic therapy (n = 1402)

Ophthalmologic examination findings	Single antithombotic therapy n (%)	Double antithrombotic therapy n (%)	No antithrombotic therapy n (%)	n (%)
Severe				
Periocular hematoma	67 (4.71%)	2 (0.14%)	343 (24.14%)	412 (28.99%)
Hyposphagma	40 (2.81%)	3 (0.21%)	222 (15.62%)	265 (18.65%)
Periorbital swelling	38 (2.67%)	3 (0.21%)	174 (12.24%)	215 (15.13%)
Eye motility disorder	22 (1.55%)	1 (0.07%)	136 (9.57%)	159 (11.19%)
Diplopia	23 (1.62%)	1 (0.07%)	115 (8.09%)	139 (9.78%)
Chemosis	17 (1.20%)	0	51 (3.59%)	68 (4.79%)
Pupil difference	1 (0.07%)	0	20 (1.41%)	21 (1.48%)
Subjective eye pain	2 (0.14%)	0	17 (1.20%)	19 (1.34%)
Ptosis	4 (0.28%)	0	10 (0.70%)	14 (0.99%)
Emphysema	3 (0.21%)	0	11 (0.77%)	14 (0.99%)
Increased intraocular pressure	2 (0.14%)	0	11 (0.77%)	13 (0.91%)
Corneal abrasion	0	0	12 (0.84%)	12 (0.84%)
Enophthalmos	1 (0.07%)	0	8 (0.56%)	9 (0.63%)
Visus acuity change	1 (0.07%)	0	8 (0.56%)	9 (0.63%)
Macular edema	1 (0.07%)	0	7 (0.49%)	8 (0.56%)
Exophthalmos	2 (0.14%)	0	4 (0.28%)	6 (0.42%)
Ectropion Optic disc hemorrhage*	1 (0.07%) 3 (0.21%)	0 0	4 (0.28%) 1 (0.07%)	5 (0.35%) 4 (0.28%)
Anterior uveitis	0	0	2 (0.14%)	2 (0.14%)
Nystagmus	0	0	1 (0.07%)	1 (0.07%)
Visual field restriction	0	0	1 (0.07%)	1 (0.07%)
Entropiom	0	0	1 (0.07%)	1 (0.07%)
Lagophthalmos	0	0	1 (0.07%)	1 (0.07%)
Pupillary swelling	1 (0.07%)	0	0	1 (0.07%)
Optic nerve congestion	0	0	1 (0.07%)	1 (0.07%)
Hyphema Non-severe Lid tear Open globe injury Retrobulbar hematoma Macular hemorrhage Foreign body Lens subluxation	0 0 0 1 (0.07%) 2 (0.14%) 0 0	0 0 0 0 0 0 0	1 (0.07%) 6 (0.42%) 4 (0.28%) 2 (0.14%) 1 (0.07%) 3 (0.21%) 1 (0.07%)	1 (0.07%) 6 (0.42%) 4 (0.28%) 3 (0.21%) 3 (0.21%) 3 (0.21%) 1 (0.07%)
n (%)	232 (16.33%)	10 (0.70%)	1179 (82.97%)	1421 (100%)

*n* = number; *%* = percentage

*Fisher’s Exact Test: **p = 0.0171**: Optic disc hemorrhage occurred significantly more frequently in patients with single antithrombotic therapy

Table 6 presents the results of multimodal bivariate analysis to measure the association between the study predictors and the severity of OPI. Specifically, blows from a blunt object and Le Fort fractures showed a significantly higher tendency towards severe OPIs (Table 6).

**Table 6:** Bivariate analysis of the study predictors and OPIs

	OPI			Ratio
	Non-severe	Severe	p value*	
	1401	20		
Etiology of injury			0.01149	
Ground-level fall	426	6		0.0138
Fall from height	65	0		0
Staircase fall	61	0		0
Road traffic accident	76	2		0.0256
Bicycle accident	251	4		0.0156
Blow from blunt object	75	5		0.0625
Violence	309	1		0.0032
Sport	89	0		0
Others	49	2		0.0392
Fracture patterns			0.02349	
Zygomatic fracture without orbital floor involvement	144	1		0.0068
Zygomatic fracture with orbital floor involvement	650	8		0.0121
Isolated orbital fractures	462	4		0.0085
Le Fort fractures	145	7		0.0460
Pre-traumatic ophthalmologic conditions			0.3218	
Glaucoma	9	1		
Cataract	76	1		
Previous eye surgery	33	0		
Glaucoma + cataract	1	0		
Claucoma + previous eye surgery	2	0		
Cataract + previous eye surgery	3	0		
Antithrombotic therapy			1	
Single medication	229	3		
Double medication	10	0		
No medication	1162	17		

*OPI* ocular and periocular injuries, *n* number, *%* percentage

*Fisher’s Exact Test with Monte-Carlo-simulation

## Discussion

We aimed to determine the incidence and types of OPIs associated with various fracture patterns of the ZMO complex. Additionally, we aimed to identify patient- and trauma-related variables that contribute to the severity of OPIs.

To address whether an ophthalmological consultation is always necessary in an emergency setting, we found that patients who experienced blows from blunt objects and those with Le Fort fractures are at higher risk of severe OPI and may require immediate ophthalmic intervention. There are some districtions between our study and others regarding ocular injuries in cases of ZMO fractures. Firstly, our clinic’s interdisciplinary approach allows for specialized ophthalmological consultation within 24 h of admission, which can prevent delays that might impact injury assessment and classification. This is crucial as a non-severe injury left untreated could potentially escalate into a severe injury [9]. In line with Oliver et al., we categorized eye injuries based on the need for urgent intervention, distinguishing between non-severe and severe injuries [9]. We deemed an eyelid tear as severe, necessitating immediate repair, while other studies suggested a secondary treatment approach [9]. However, our study differed from Oliver et al. as our sample included patients with various fracture patterns of the ZMO complex, not solely orbital fractures. Furthermore, unlike Oliver et al. and Chow et al., our reported rate of severe ocular injury (1.4%) was relatively low, consistent with previous research findings [9, 19–25].

Regarding the study population, the average patient age was slightly above the range reported in the literature, with an average between 42 and 47.4 years [1, 11, 12, 20, 21]. Similar to our findings, previous studies have also shown higher rates of injuries among male patients (67.7–87.7%) [1, 11, 12, 20, 22–27]. In terms of the cause of trauma, previous research indicates that traffic accidents are the most common cause, followed by tripping falls and violence [4, 10, 21, 25, 28–31]. However, in our study, tripping falls were the most frequently recorded cause. Contrary to our results, Al-Qurainy et al. reported that 20% of traffic accidents and 11% of violence cases were linked to severe ocular injuries [32]. On the other hand, Zhong et al. argued that the mechanism of trauma did not impact the severity of eye injury. These discrepancies may be attributed to various sociodemographic differences on an international scale [8].

Regarding the fracture pattern, most available data describe ocular injuries following orbital wall fractures [9]. We documented zygomatic fractures with orbital involvement as the most frequent, followed by isolated orbital wall fractures. In isolated orbital wall fractures, the floor was as expected the most commonly affected, followed by the medial wall, the lateral wall, and the orbital roof, respectively. This distribution pattern is similarly described in the studies of Committeri et al. and Zhong et al. [1, 8]. Similar to previous studies, we observed higher OPI rates in patients with fractures of the ZMO complex [8, 22, 23, 29, 33]. However, other authors have reported ocular involvement more frequently in isolated orbital wall fractures [23, 33, 34]. Unlike Oliver et al., we found no association between isolated orbital fractures and severe OPIs. The location of the orbital wall fractures was also not associated with the ophthalmologic findings. Our results are in concordance with Noh et al. [35]. We agree with Mangan et al. that the extent of orbital fracture, type of tissue prolapse and the presence of muscle entrapment could provide further clarity in identifying key fracture details related to specific ocular injuries [36]. However, this was not the aim of our study. The complexity of the relationship between orbital fracture patterns and OPI results in a lack of consensus on which fracture pattern or location has the highest risk of OPI, and further research is needed to address this subject of debate [9, 37–39].

According to current literature, the likelihood of OPI after midface trauma ranges from 3.9 to 95.7% [1, 4, 12, 21–24, 28, 29, 31, 32, 34, 40–46]. These variations in rates can be explained by different fracture patterns and specific post-traumatic ocular findings that were evaluated individually. We found that periocular hematomas were the most common post-traumatic finding, followed by hyposphagma and periorbital swelling. This distribution was consistent across all age groups and aligns with previous studies [3, 4, 11, 12, 20, 21, 23–25, 27, 29–31, 43].

In contrast to the studies of Andrews et al. and Zhong et al., we reported significant associations between certain ocular findings and fracture patterns [8, 22]. Patients with an isolated orbital wall fracture suffered significantly more frequently from motility disorders and diplopia, in line with previous research [1, 4, 20, 47]. The higher probability of damage to the eye muscle attachments due to the anatomical proximity to the orbital walls leading to a change in position and alignment of the globe could explain this finding. Chemosis also occurred significantly more frequently in fractures of the ZMO complex, which may be due to the direct impact on a larger, well-perfused soft and bone tissue area following this fracture pattern compared to Le Fort or isolated orbital or zygomatic fractures. There is not much evidence regarding ophthalmologic pathologies after Le Fort fractures. We have demonstrated that Le Fort fractures are more likely to result in severe OPI. However, it is important to note that a valid statement cannot be made due to the limited sample size of this fracture pattern and the lack of similar studies for comparison [31].

No previous study has investigated the potential correlation between pre-traumatic ophthalmologic conditions such as cataract or glaucoma and post-traumatic ophthalmologic findings. While we observed hyposphagma, eye motility disorders and diplopia more frequently in patients with these pre-traumatic conditions, there was no statistically significant correlation. Blumer et al. examined the effects of previous eye surgery on post-traumatic ophthalmologic findings in 28 cases, reporting hyposphagma, diplopia, chemosis and eye bruising as the most common symptoms, but without significant relevance [20]. Similarly, our results suggest that previous eye surgery is not associated with a higher risk of ocular injury. Further prospective research is needed to provide more reliable insights into significant correlations.

To date, there is no valid evidence regarding the impact of antithrombotic medication on the risk of significant ophthalmologic injuries after midface trauma. Due to the small sample size, only the different antithrombotic groups, not the individual anticoagulant substances could be statistically analyzed for significance. We found that optic disc hemorrhage occurred significantly more frequently in patients under single antithrombotic therapy; however, no specific antithrombotic groups were identified as riskier. Despite this, a significantly higher risk of OPI in patients under single or double antithrombotic therapy or among the different medication groups and substances could not be demonstrated. Only Zhong et al. evaluated the effect of anticoagulant medication on the risk of severe ocular hemorrhage in patients with orbital wall fractures [8]. They concluded that the use of aspirin, apixaban, warfarin, ticagrelor and clopidogrel did not significantly contribute to severe ophthalmologic injury or severe ocular hemorrhage. Further studies with a larger sample size could validate our findings and explore the potential correlation of antithrombotic therapy with the risk of severe post-traumatic OPI.

Considering the current controversy surrounding the necessity of ophthalmologic evaluation for every patient with ZMO fractures, we aimed to identify risk groups for whom a primary ophthalmological examination should be considered earlier in the emergency setting. Our results do not support previous studies that recommend a specialized ophthalmological examination within the first 24 h for every patient with orbital/midfacial trauma [3, 7]. However, we recommend conducting a thorough history of the trauma mechanism to assess the severity of the fracture and associated OPI. The initial clinical examination should include inspection, palpation, assessment of neurological status, and a basic ocular examination performed by a maxillofacial surgeon as soon as possible, followed by CT imaging. We recommend early ophthalmological consultation for patients with more complicated fracture patterns such as Le Fort fractures and patients under antithrombotic therapy, even if visual acuity is preserved at presentation to avoid missing intraocular pathologies with risks to optic nerve function due to secondary hemorrhage. Cohen et al. stated that normal visual acuity at presentation does not guarantee absence of ocular injury [48]. While maxillofacial surgeons can assess bulbus motility and visual acuity disorders as part of the initial examination to gain an initial impression of ophthalmic involvement, minor ocular injuries such as macular edema or retinal hemorrhage might go unnoticed. In cases where there is suspicion of acute eye injury, severe eye pain, sudden vision loss, motility issues, or visible bulbar injuries, a specialized ophthalmologic assessment should rule out retrobulbar hematoma, intraocular hemorrhage and any retinal detachment, tears or lens subluxation before proceeding to the fracture management. Additionally, preoperatively assessing and documenting ocular injuries is crucial from a medicolegal perspective to ensure that any postoperative visual changes are not attributed to surgery [2].

There are some limitations to the current study. The retrospective nature of this observational research could lead to documentation bias. However, this limitation is outweighed by the large patient collective, which allowed the comparison of various fracture patterns regarding the risk of an ophthalmologic injury. Secondly, the ophthalmological examination findings were recorded by different ophthalmologists with varying levels of training and experience, which could lead to observer bias. Third, depending on the examiner and the time of examination, the findings in the medical records were sometimes described in more or less detail. The lack of documented visual acuity or intraocular pressure at the time of presentation, mostly explained due to the emergency setting, can lead to documentation bias regarding the exact ophthalmic health. Additionally, an exact time of the initial examination was not always documented, thus no valid statement can be made about the best time point for the ophthalmological evaluation. Fourth, not performing a dilated fundus examination in cases of closed-globe injury is also acknowledged as a limitation. Fifth, since every patient presented various findings at the same time, many subgroups resulted in a small number of patients. The collective inhomogeneity due to the small number of patients in the subgroups may also lead to bias by underestimating a specific fracture pattern or a specific antithrombotic substance as a real risk factor for severe ocular injury. This could limit the generalizability of our results. Sixth, only a few patients with previous ophthalmological conditions such as cataract and glaucoma could be included, and the grade of the disease was not documented sufficiently. This could limit the conclusions since advanced cataract and glaucoma with blindness may have a higher propensity for leading to trauma. In future research, a larger group of patients with a variety of previous pathologies should be examined for more reliable statistical statements. Additionally, other factors such as previous ocular trauma or hypertension can also be taken into account. Lastly, the lack of follow-up data can limit the clinical relevance of the results. Future prospective studies with standardized observation protocols are needed to understand the complex relationship between ZMO fractures, associated conditions and OPIs.

## Conclusion

Within the limitations of the study, the results suggest that patients who experienced blows from blunt objects and those with Le Fort fractures are at a higher risk for severe OPIs. We recommend early specialized ophthalmological consultation for patients with zygomatic fractures and orbital involvement, especially for those with visual alterations such as motility disorders and diplopia as well as those taking antithrombotic medication. This could prioritize the referral of patients to an ophthalmologist, particularly in hospitals lacking ophthalmology departments to ensure the best post-traumatic outcome and prevent complications and legal issues. Future prospective studies with standardized observation protocols are needed to validate these recommendations for clinical practice.

## Data Availability

The datasets used and/or analysed during the current study available from the corresponding author on reasonable request.
